# Zinc ions have a potential to attenuate both Ni ion uptake and Ni ion-induced inflammation

**DOI:** 10.1038/s41598-018-21014-8

**Published:** 2018-02-13

**Authors:** Ryo Onodera, Sanki Asakawa, Ryosuke Segawa, Natsumi Mizuno, Kouetsu Ogasawara, Masahiro Hiratsuka, Noriyasu Hirasawa

**Affiliations:** 1 Laboratory of Pharmacotherapy of Life-Style Related Diseases, Graduate School of Pharmaceutical Sciences, Tohoku University, Sendai, Miyagi 980-8578 Japan; 2 Laboratory of Immunobiology, Institute of Development, Aging, and Cancer, Tohoku University, Sendai, Miyagi 980-8575 Japan

## Abstract

Nickel ions (Ni^2+^) are eluted from various metallic materials, such as medical devices implanted in human tissues. Previous studies have shown that Ni^2+^ enters inflammatory cells inducing inflammation. However, the regulation of Ni^2+^ uptake in cells has not yet been reported in detail. In the present study, we investigated the effects of various divalent cations on Ni^2+^ uptake and Ni^2+^-induced interleukin (IL)-8 production in the human monocytic cell line, THP-1. We demonstrated that ZnCl_2,_ MnCl_2_, and CoCl_2_ inhibited the Ni^2+^ uptake, while CuCl_2_, FeCl_2_, MgCl_2_, and divalent metal transporter (DMT)-1 inhibitor, Chlorazol Black, did not. Furthermore, ZnCl_2_ inhibited Ni^2+^-induced IL-8 production, correlating with the inhibition of Ni^2+^ uptake. These results suggested that Ni^2+^ uptake occurred through Zn^2+^, Mn^2+^, and Co^2+^-sensitive transporters and that the inhibition of Ni^2+^ uptake resulted in the inhibition of IL-8 production. Furthermore, using an Ni wire-implanted mouse model, we found that Ni wire-induced expression of mouse macrophage inflammatory protein-2 (MIP-2) and cyclooxygenase-2 (COX-2) mRNA in the skin tissue surrounding the wire were enhanced by low Zn conditions. These results suggested that the physiological concentration of Zn^2+^ modulates Ni^2+^ uptake by inflammatory cells, and a Zn deficient state might increase sensitivity to Ni.

## Introduction

Nickel (Ni) is included in several medical devices, including prostheses, pace makers, stents, and dental implants, owing to its beneficial properties such as resistance to corrosion and durability. However, Ni ion elutes from Ni-containing materials possibly causing inflammation^1–3^. Actually, the prevention of neointima formation by Ni-free stainless stent was demonstrated^4^. We also reported that the implantation of an Ni wire subcutaneously into the back of mice induced the elution of Ni^2+^, the expression of several inflammatory proteins such as cyclooxygenase-2 (COX-2) and neutrophil chemokine macrophage inflammatory protein-2 (MIP-2, CXCL2), and leukocyte infiltration as the initial responses^5,6^. Importantly, infiltration and activation of neutrophils enhanced further elution of Ni^2+^^5^. Thus, inhibition of Ni^2+^-induced inflammatory cell activation would be one of the strategies to prevent Ni^2+^ elution.

It was generally accepted that Ni^2+^ binds to various extracellular proteins to form a novel antigen causing delayed-type hypersensitivity^7–9^. For example, Ni^2+^ binds to human serum albumin inducing activation of human T cells^9^. Furthermore, Ni^2+^ forms different Ni epitopes leading to polyclonal Ni-specific T cell activation. However, Ni^2+^ directly activates various inflammatory cells^5^ and induces death of monocytes^10^. For example, Ni^2+^ binds to Toll-like receptor 4 (TLR4) on the cell surface, activating the NF-κB pathway^11^. In addition to cell surface proteins, Ni^2+^ binds to and modulates intracellular proteins; these ions enter the cells and inhibit prolyl hydroxylases (PHDs), resulting in the activation of a transcription factor called the hypoxia-inducing factor-1α (HIF-1α)^4,12^. As HIF-1α activation plays crucial roles in cytokine production and angiogenesis, Ni^2+^ uptake into the cells was one of the important steps in Ni^2+^-induced damage.

Transporters for Ni^2+^ uptake have been reported in microorganisms^13,14^. In contrast, Ni^2+^ transport systems in human cells have not yet been identified. The uptake of heavy metal ions, such as Cu^2+^, Fe^2+^, and Zn^2+^, occurs via the divalent metal transporter, DMT1, in mammalian cells^15,16^. The Zn transporter, Zrt- and Irt-like protein (ZIP, SLC39A) family, which consists of over 25 members^17^, is also involved in the influx of several heavy metal ions. Each of these members exhibits specificity toward a specific metal. However, the metal specificity of the transporter involved in Ni^2+^ uptake remains unclear.

Ni^2+^ uptake in cells and nuclei in the human monocytic cell line, THP-1, has already been reported^18^. THP-1 cells also have the ability to produce IL-8 by treatment with Ni compounds^19,20^. Therefore, using THP-1 cells, we examined whether the competition between Ni^2+^ and other ions affected IL-8 production. Especially, to assess the accumulation of metals in the cells and Ni^2+^ elution in the tissues precisely, we used inductively coupled plasma mass spectrometry (ICP-MS), a highly sensitive and efficient analysis technique for detecting various metal ions. In this study, we found that the physiological concentration of Zn^2+^ affected the uptake of Ni^2+^ by THP-1 cells and the sensitivity of mouse to Ni^2+^.

## Results

### NiCl_2_-stimulated increase in Ni^2+^ content and IL-8 production in THP-1 cells

**Figure 1:**
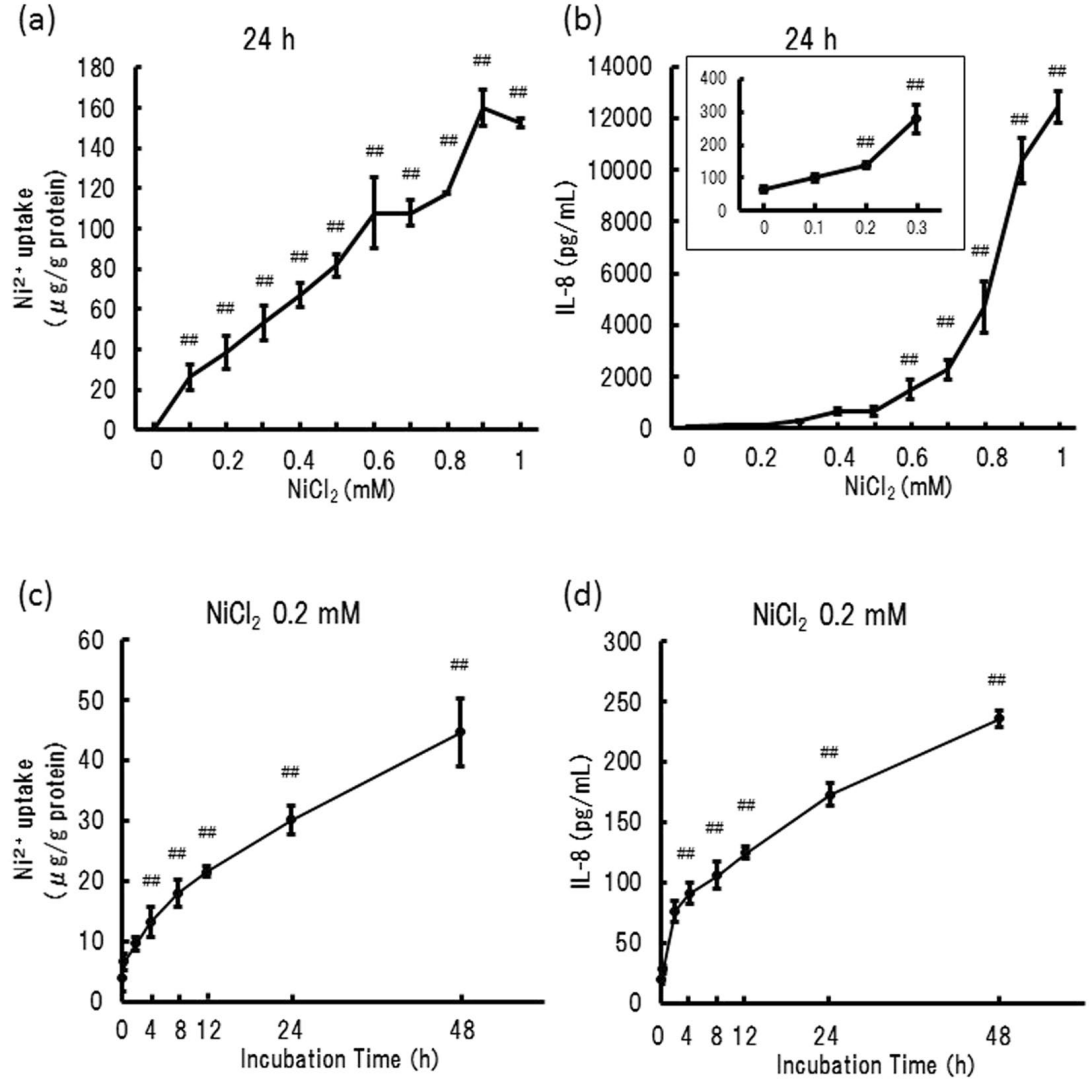
Ni^2+^ uptake and IL-8 production in THP-1 cells. THP-1 cells were treated with various concentrations of NiCl_2_ for 24 h (**a** and **b**) and 0.2 mM NiCl_2_ for the indicated times (**c** and **d**). The amount of Ni^2+^ in the cells (**a** and **c**) and IL-8 in the supernatant (**b** and **d**) were determined using ICP-MS and ELISA, respectively. The vertical lines represent the S.E.M. of 3 samples. ^##^*p* < 0.01 vs. 0 mM (**a** and **b**) or 0 h (**c** and **d**).

THP-1 cells were treated with various concentrations of NiCl_2_ for 24 h and the Ni^2+^ content in the cells and IL-8 level in the medium were determined. Both Ni^2+^ content and IL-8 production increased in a NiCl_2_ concentration-dependent manner (Fig. 1a and b). As IL-8 production was significantly induced by NiCl_2_ at the concentration of ≥0.2 mM (Fig. 1b), 0.2 mM NiCl_2_ was used in all the experiments. Ni^2+^ content in the cells increased in a time-dependent manner (Fig. 1c), consistent with the concentration-dependent increase in the cells, and IL-8 level in the medium increased significantly from the 4-h mark (Fig. 1d). The incubation of THP-1 cells in 0.2 mM NiCl_2_ for 24 h did not affect the viability as determined by the MTT assay (data not shown).

### Effects of metal ions on the uptake of Ni ions

**Figure 2:**
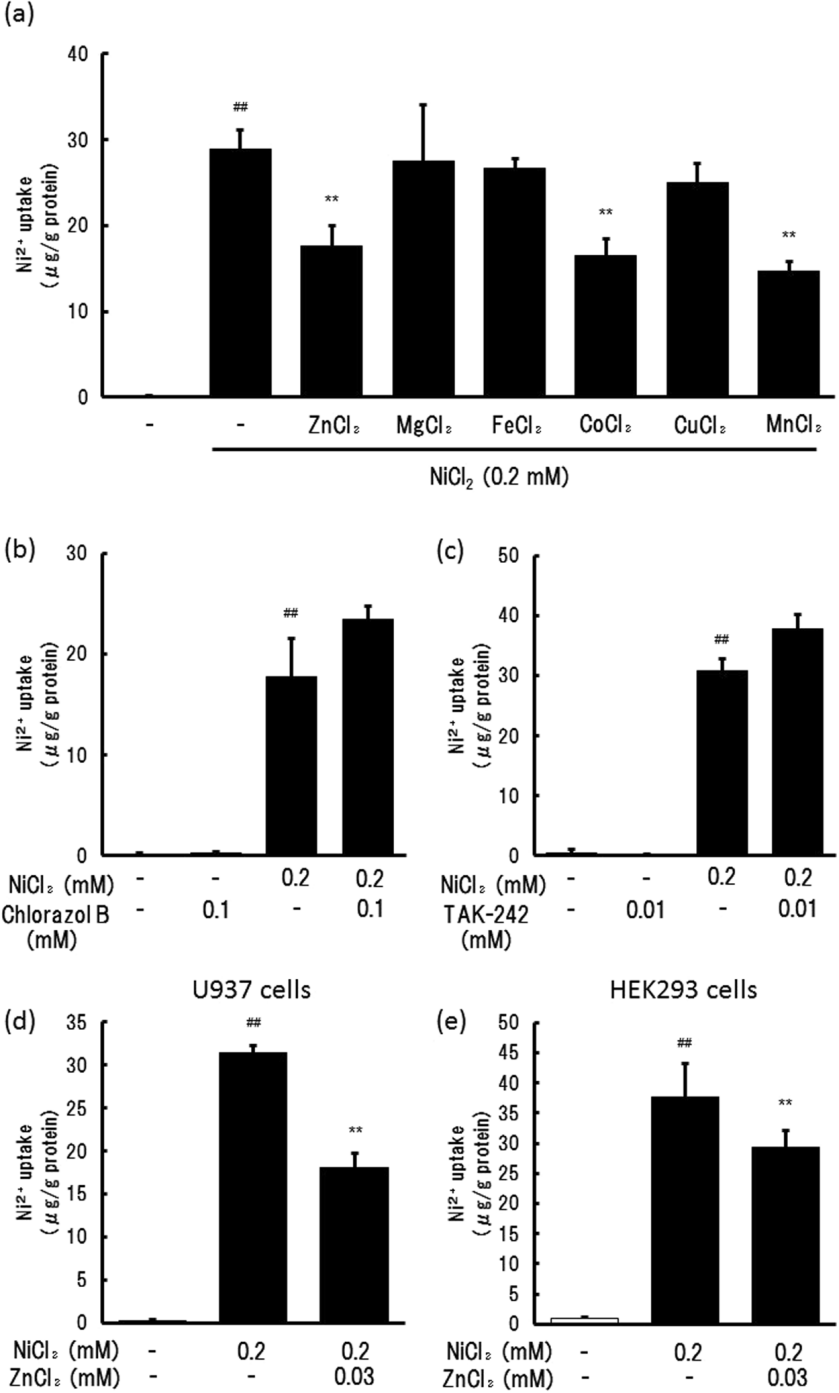
Effects of divalent cations and inhibitors on Ni^2+^ uptake. (**a**,**b**, and **c**): THP-1 cells were treated with 0.2 mM NiCl_2_ in the presence or absence of 0.03 mM metal chlorides (**a**), 0.1 mM Chlorazol B (**b**), or 0.01 mM TAK-242 (**c**) for 24 h. (**d** and **e**): U937 (**d**) and HEK293 (**e**) cells were treated with NiCl_2_ in the presence or absence of 0.03 mM ZnCl_2_ for 24 h. The Ni^2+^ uptake of the cells was determined using ICP-MS. The vertical lines represent the S.E.M. of 3 samples. ^##^*p* < 0.01 vs. Control, **p < 0.01 vs. 0.2 mM NiCl_2_.

THP-1 cells were treated with 0.2 mM NiCl_2_ in the presence of various divalent cations (0.03 mM), including Zn^2+^, Mg^2+^, Fe^2+^, Co^2+^, Cu^2+^, or Mn^2+^, added as dichloride salts. The Ni^2+^ content in the cells after 24 h of incubation was determined by ICP-MS. The increase in the intracellular Ni^2+^ content was inhibited by ZnCl_2_, CoCl_2_, and MnCl_2_ (Fig. 2a). In contrast, the increase in Ni^2+^ content was not inhibited by the divalent metal transporter 1 (DMT1) inhibitor, Chlorazol Black (Fig. 2b). Because Ni^2+^ activates Toll-like receptor 4 (TLR4), the effects of the TLR4 inhibitor, TAK-242, on Ni^2+^ uptake were determined. The results suggested that TAK-242 did not affect Ni^2+^ uptake (Fig. 2c), suggesting that TLR4 activation was not involved in Ni^2+^ uptake. To confirm whether ZnCl_2_ also inhibits Ni^2+^ uptake in the other cell lines, a human monocytic cell line, U937 (Fig. 2d), and a human embryonic kidney cell line, HEK293 (Fig. 2e) were treated with 0.2 mM NiCl_2_ in the presence of 0.03 mM ZnCl_2_. Ni^2+^ content in these cells was increased by NiCl_2_ treatment, and this increase was reduced by ZnCl_2_. These findings suggested that Ni^2+^ uptake occurred generally via a Zn^2+^-sensitive transporter.

### Cellular compartmentalization of Ni ions and the effects of ZnCl_2_

**Figure 3:**
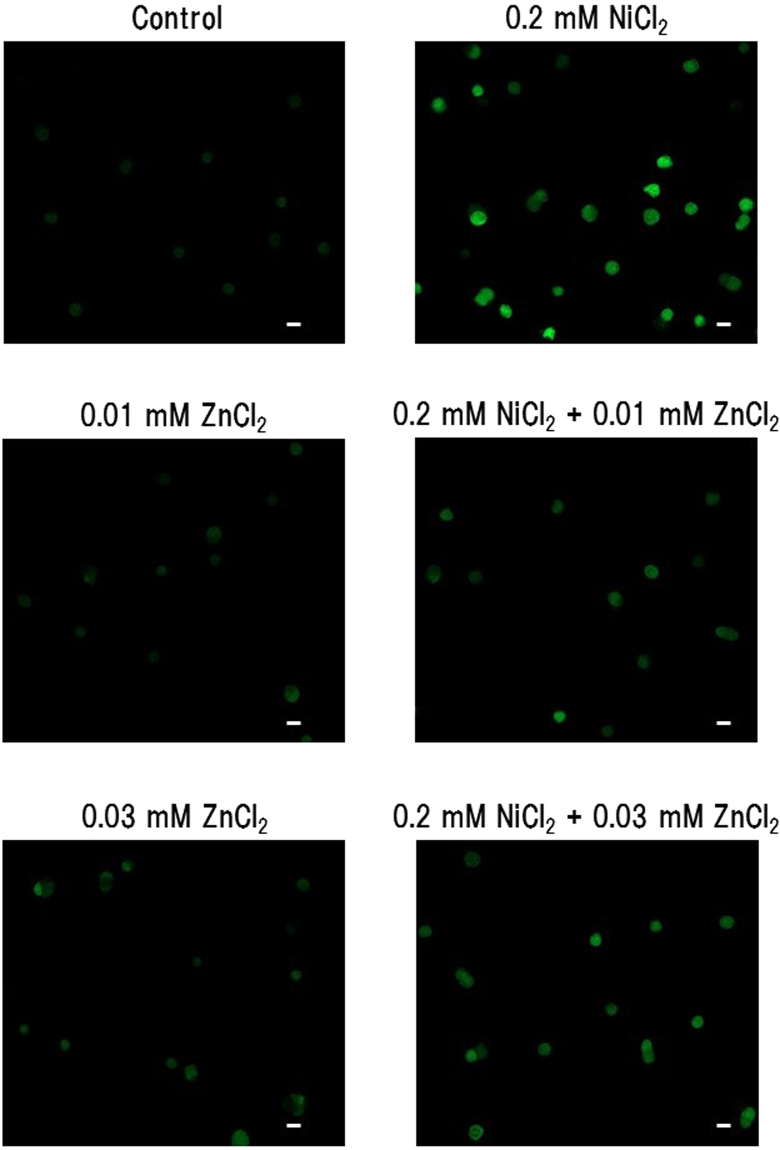
Detection of Ni^2+^ in the cells by Newport Green. THP-1 cells were treated with 0.2 mM NiCl_2_ in the presence or absence of 0.01 and 0.03 mM ZnCl_2_ for 24 h. Intracellular Ni^2+^ content was detected with Newport Green. The white scale bar indicates 10 μm.

To confirm whether Ni^2+^ entered the cells or was bound to the cell membrane, the cellular compartmentalization of Ni^2+^ was determined by the fluorescence indicator, Newport Green. This compound was used to detect Ni^2+^ in the immune cells in a previous study^21^. Although Newport Green could bind to both Zn^2+^ and Ni^2+^, the concentration of ZnCl_2_ used in this experiment, 0.01 mM, did not apparently increase the fluorescence. In contrast, treatment with 0.2 mM NiCl_2_ increased the fluorescence in the cells, indicating that Ni^2+^ entered the cells. Consistent with the data of ICP-MS, treatment with ZnCl_2_ inhibited the NiCl_2_-induced increase in fluorescence (Fig. 3), indicating that even at a low concentration, Zn^2+^ inhibited Ni^2+^ uptake.

### Effects of ZnCl_2_ and MnCl_2_ on Ni^2+^-induced IL-8 production

**Figure 4:**
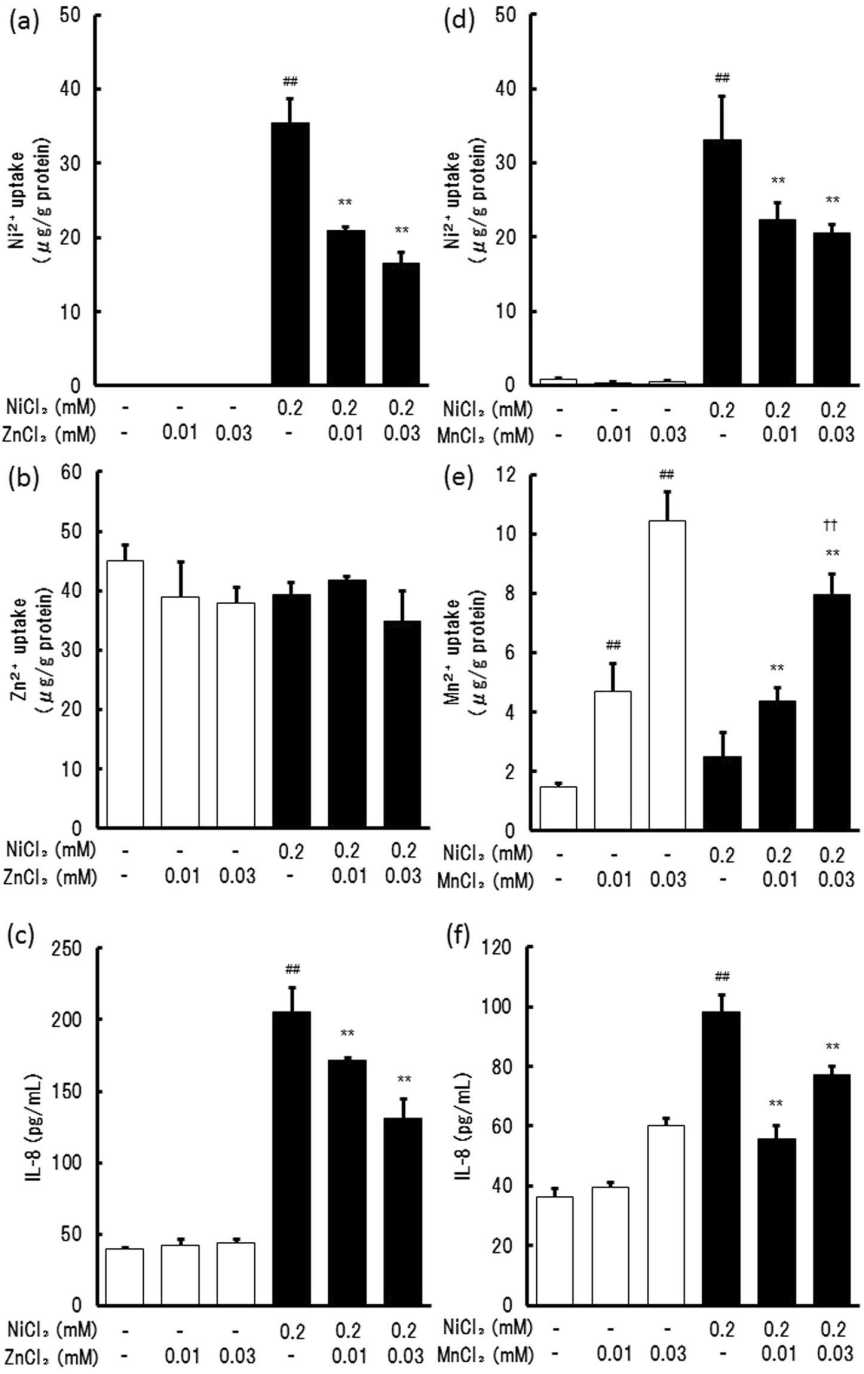
Effect of ZnCl_2_ or MnCl_2_ on Ni^2+^ uptake and IL-8 production in THP-1 cells. THP-1 cells were treated with NiCl_2_ in the presence of 0.01 and 0.03 mM ZnCl_2_ (**a**–**c**) or MnCl_2_ (**d**–**f**) for 24 h and then the amounts of Ni^2+^ (**a** and **d**), Zn^2+^ (**b**), and Mn^2+^ (**e**) in the cells, and IL-8 in the supernatant (**c** and **f**) were determined using ICP-MS and ELISA, respectively. The vertical lines represent the S.E.M. of 3 samples. ^##^*p* < 0.01 vs. Control, **p < 0.01 vs. 0.2 mM NiCl_2_, ^††^*p* < 0.01 vs. 0.03 mM MnCl_2_.

To clarify whether the inhibition of Ni^2+^ uptake resulted in the inhibition of IL-8 production, the cells were treated with 0.2 mM NiCl_2_ in the presence of 0.01 and 0.03 mM ZnCl_2_ and MnCl_2_. The increase in the Ni^2+^ content was reduced by ZnCl_2_ and MnCl_2_ in a concentration-dependent manner (Fig. 4a and d). Treatment with ZnCl_2_ did not affect the Zn^2+^ content in the cells, but that with MnCl_2_ increased the Mn^2+^ content. In these conditions, IL-8 production was also inhibited by these cations (Fig. 4c and f). MnCl_2_ at 0.03 mM concentration slightly induced IL-8 production by itself, both in the presence and absence of NiCl_2_ (Fig. 4f), indicating that Mn^2+^ has a weak ability to induce IL-8 production by itself.

### Effects of ZnCl_2_ on CoCl_2_- and LPS-induced IL-8 production

**Figure 5:**
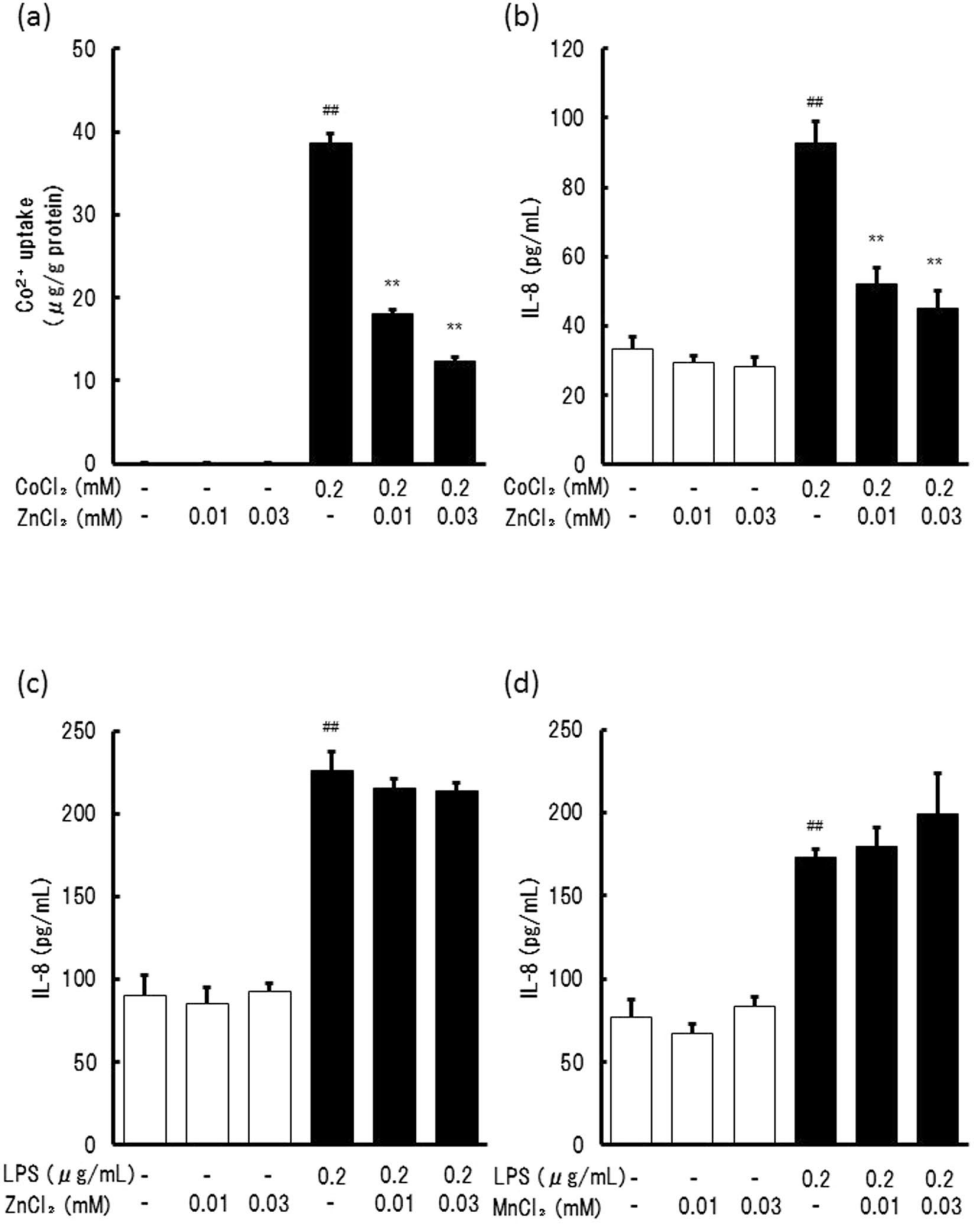
Effects of ZnCl_2_ on Co^2+^ uptake and IL-8 production induced by CoCl_2_ and LPS. THP-1 cells were treated with CoCl_2_ (**a** and **b**) and 0.2 μg/ml LPS (**c** and **d**) in the presence of 0.01 and 0.03 mM ZnCl_2_ for 24 h. The amounts of Co^2+^ (**a**) in the cells, and IL-8 in the supernatant (**b**,**c**, and **d**) were then determined using ICP-MS and ELISA, respectively. The vertical lines represent the S.E.M. of 3 samples. ^##^*p* < 0.01 vs. Control, **p < 0.01 vs. 0.2 mM CoCl_2_.

To confirm the selectivity of the action of ZnCl_2_, the effects of ZnCl_2_ on CoCl_2_- and LPS-induced IL-8 production were examined. Treatment with 0.2 mM CoCl_2_ increased Co^2+^ content in the cells and IL-8 production. ZnCl_2_ (0.01 and 0.03 mM) inhibited this increase in a dose-dependent manner (Fig. 5a and b). In contrast, the same concentrations of ZnCl_2_ and MnCl_2_ did not inhibit LPS-induced IL-8 production (Fig. 5c and d), indicating that Zn^2+^ did not affect the signaling pathway inducing IL-8 expression in this case.

### Enhancement of Ni wire-induced inflammation in a Zn-deficient state

**Figure 6:**
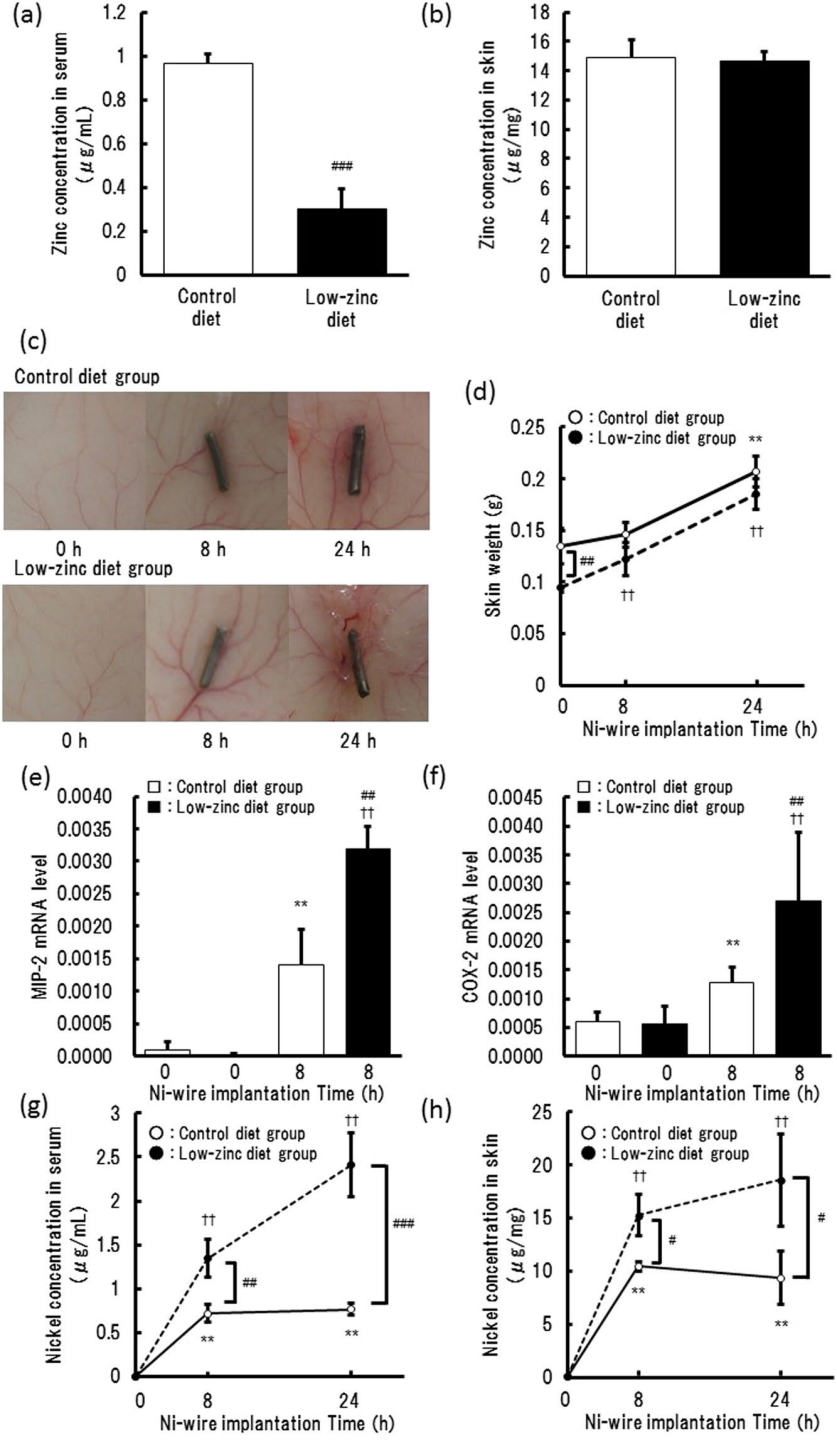
Enhancement of Ni^2+^-induced inflammation in a Zn-deficient state in mice. Mice were fed a low-Zn diet or normal diet for 2 weeks and then an Ni wire was implanted subcutaneously in their dorsa. The mice were sacrificed 0, 8, or 24 h after the implantation. The amounts of Zn^2+^ in the serum (**a**) and skin (**b**) of mice before the implantation were determined using ICP-MS. The skin around the wire was photographed (**c**) and weighed (**d**). Ni^2+^ in the serum (**g**) and skin (**h**) were determined using ICP-MS. The expression of MIP-2 (**e**) and COX-2 (**f**) was measured by qRT-PCR for the respective times. Values are normalized to those of GAPDH. The vertical lines represent the S.E.M. of the respective values for 3–4 mice. **p < 0.01 vs. 0 h control diet group, ^#^*p* < 0.05, ^##^*p* < 0.01, ^###^*p* < 0.001 vs. the corresponding control diet group, ^††^*p* < 0.01 vs. 0 h low-Zn diet group.

Finally, we examined whether the physiological concentration of Zn^2+^ affects Ni^2+^-induced inflammation in low Zn diet-fed mice. Consumption of the low-Zn diet for two weeks reduced Zn^2+^ levels in the serum to one third of the normal levels (Fig. 6a), but the level in the skin tissues was unchanged (Fig. 6b). As previously reported, implantation of the Ni wire on the back of mice induced inflammation, visible as vasodilation/erythema (Fig. 6c), edema (Fig. 6d), and the expression of inflammatory proteins such as MIP-2 (Fig. 6e) and COX-2 (Fig. 6f). In the mice fed with low-Zn diet for 2 weeks, interestingly, the Ni^2+^-induced expression of MIP-2 and COX-2 was significantly higher than that in the control group (Fig. 6e and f). The concentration of Ni^2+^ in the serum and skin tissues was also higher in the low-Zn diet group than in the control group (Fig. 6g and h), indicating that enhanced inflammation promoted Ni^2+^ elution.

## Discussion

In this study, we found that Ni^2+^ entered the THP-1 cells in a Zn^2+^, Mn^2+^, and Co^2+^-sensitive manner, and that Zn^2+^ inhibited Ni^2+^ uptake, resulting in reduced IL-8 production. More importantly, we showed that Ni^2+^-induced inflammation was enhanced in a systemic low-Zn state. Our findings suggest that maintaining a normal level of Zn^2+^ is important to reduce the incidence of Ni-induced inflammation and allergy.

As expected, the incubation of THP-1 in the presence of NiCl_2,_ elicited an increase in intracellular Ni^2+^ level and IL-8 production. The accumulation of Ni^2+^ in THP-1 cells was induced rapidly until 4 h and then it accumulated gradually. The findings, consistent with those in the previous report^18^, suggested that the increase was regulated by Ni^2+^ influx and efflux balance. The increase in Ni^2+^ level in the cells was antagonized by Zn^2+^, Mn^2+^, and Co^2+^, indicating the involvement of transporter(s) sensitive to these divalent cations. The antagonizing effects of ZnCl_2_ and MnCl_2_ were observed at concentrations lower than those of NiCl_2_, indicating that the affinity of Zn^2+^ and Mn^2+^ was much higher than that of Ni^2+^ to the transporter. The putative transporters were DMT1 and ZIPs. Although DMT1 has an affinity to Ni^2+^^16^, it was likely to contribute minimally to Ni^2+^ uptake in THP-1 cells, because the DMT1 inhibitor, Chlorazol Black^22,23^, did not decrease Ni^2+^ uptake. The ZIP family consists of several members and some of them have an affinity to Ni^2+^^24–26^. All ZIPs except for ZIP12 were expressed in THP-1 cells^27^, and ZIP2^25,28^, ZIP3^26^, ZIP8, and ZIP14^24,29,30^ have been shown to have an affinity to Zn^2+^, Mn^2+^, Co^2+^. In addition, ZIPs are known to be induced by the stimulation of TLR4^31^. However, the possibility that Ni^2+^ induced Zn transporters via the stimulation of TLR4 was rejected, because TAK-242 did not affect the increase in Ni content in the cells incubated for 24 h. These findings suggested that the Ni^2+^ entered via constitutively expressed ZIP-type transporters. However, because several family members might be involved in Ni^2+^ uptake and because they have no specific inhibitors, it was difficult to identify the one responsible in this case. We started screening the specific inhibitors of Ni^2+^ influx to identify the transporter.

We, for the first time, also showed that antagonizing Ni^2+^ uptake by Zn^2+^ resulted in the inhibition of IL-8 production. Zn^2+^ also inhibited Co^2+^ uptake and Co^2+^-induced IL-8 production whereas Zn^2+^ did not inhibit LPS-induced IL-8 production, indicating that Zn^2+^ did not affect the signaling pathway for IL-8 expression. In contrast, although Mn^2+^ inhibited Ni^2+^ uptake, Mn^2+^ itself induced IL-8 production. These findings were consistent with the observation that Mn^2+^ as well as Ni^2+^ could activate HIF-1α^12^. These findings also suggested that Zn^2+^ has the ability to attenuate Ni^2+^ and Co^2+^-induced inflammation.

The protective effects of Zn^2+^ at physiological concentrations were also observed in an *in vivo* model. We had reported that Ni^2+^ elution from the Ni wire induced inflammatory events, such as neutrophil infiltration and prostaglandin and histamine production^5,6^, and that the initial inflammatory responses induced further elution of Ni^2+^^5^. Using the Ni wire-implanted mouse model, we showed that Ni^2+^-induced inflammation was enhanced in a Zn-deficient state. Additionally, the mice fed with Zn-deficient diet for 2 weeks showed an enhanced Ni wire-induced expression of MIP-2, a neutrophil chemokine, and COX-2. The elution of Ni^2+^ was also enhanced, probably via augmentation of the inflammation, as consistent with the previous study. The severe Zn deficiency causes various defects in the function of the skin, such as barrier function. However, in our condition, although Zn^2+^ concentration in the serum was apparently decreased, that in the skin was unchanged, indicating that functions of the skin were not impaired. Even though the Ni^2+^ elution and Ni^2+^-induced cytokine expression were enhanced, this suggested that the concentration of Zn^2+^ in the serum and/or in the intercellular fluids affected the Ni^2+^ uptake of leukocytes infiltrated from the blood stream. These results suggested that Ni^2+^-induced inflammatory cell responses were enhanced in the Zn-deficient state, resulting in the increase in Ni^2+^ elution. As we focused on the initial responses induced by the uptake of Ni^2+^, whether the changes in these responses affect the induction of Ni allergy remain to be elucidated. The effects of Zn-deficient condition on Ni allergy are under investigation.

The present *in vitro* and *in vivo* findings suggested that Zn^2+^ modulated Ni^2+^ uptake and the activation of the inflammatory cells. Our findings also suggested the need to issue a warning that a Zn-deficient state may exacerbate medical device-induced inflammation. A recent report indicated that the prevalence of Zn deficiency in Japanese adult males and females increased with increasing age, and that infants were also susceptible to Zn deficiency^32^. Therefore, it is important to ascertain whether people with Zn-deficiency are susceptible to Ni allergy, and to determine Zn^2+^ levels to avoid the induction of Ni-induced inflammation in people implanted with medical devices.

## Methods

Nickel chloride (NiCl_2_), zinc chloride (ZnCl_2_), cobalt chloride (CoCl_2_), copper (II) chloride dihydrate (CuCl_2_·2H_2_O), iron (II) chloride tetrahydrate (FeCl_2_·4H_2_O), magnesium chloride hexahydrate (MgCl_2_·6H_2_O), manganese (II) chloride tetrahydrate (MnCl_2_·4H_2_O), lipopolysaccharides (LPS) from *Escherichia coli* O111, and 30% (w/v) H_2_O_2_ were purchased from Wako Pure Chemical Industries (Osaka, Japan). Chlorazol Black and TAK-242 were purchased from Sigma-Aldrich Co. (St. Louis, MO) and Calbiochem-Merck Millipore (Darmstadt, Germany), respectively. Newport Green^TM^ DCF diacetate was purchased from Invitrogen (Carlsbad, CA) and the Ni wire (purity 99.98%, diameter 0.8 mm) from Nilako (Tokyo, Japan). HNO_3_ (69% (w/w)) was purchased from Kanto Chemical Co., Inc. (Tokyo, Japan).

### Cell culture

The human monocytic cell line, THP-1 (Cell Resource Center, Tohoku University) and U937 (JCRB Cell Bank, National Institute of Biomedical Innovation, Health and Nutrition, Japan), and the human epithelial cell line, HEK293 (ATCC, Manassas, VA) were used. Cells were cultured in RPMI 1640 medium (Nissui, Tokyo, Japan) supplemented with 10% (v/v) heat-inactivated fetal bovine serum (FBS, Biowest, Miami, FL), penicillin G potassium (18 μg/ml), streptomycin sulfate (50 μg/ml), L-glutamine (0.3 mg/ml), and NaHCO_3_ (1.8 mg/ml), and incubated at 37 °C under a humidified atmosphere containing 5% CO_2_.

### Mice

Four-week-old male ICR mice were purchased from SLC (Shizuoka, Japan). They were fed a standard diet (CE-2, CLEA, Tokyo, Japan) (control diet group, n = 12) or a Zn-deficient diet (CLEA, Tokyo, Japan) (low-Zn diet group, n = 12) for two weeks under a 12-h light/dark cycle in a specific, pathogen-free barrier facility. All animal experiments were approved by the Institutional Animal Care and Use Committee of Tohoku University, and performed in accordance with the Regulations for Animal Experiments and Related Activities at Tohoku University and Guidelines for Proper Conduct of Animal Experiments by the Ministry of Education, Culture, Sports, Sciences, and Technology of Japan.

### Treatment of cells with stimulants and inhibitors

NiCl_2_, ZnCl_2_, CoCl_2_, CuCl_2_, FeCl_2_, MgCl_2_, MnCl_2_, and LPS were dissolved in water. Chlorazol Black and TAK-242 were dissolved in dimethyl sulfoxide. THP-1 cells (5.0 × 10^5^ cells/ml) were seeded into 24-well plates, and stimulated with various concentrations of these reagents. The inhibitors were added with NiCl_2_.

### Implantation of the Ni wire

The Ni wire was cut into 5-mm length, sterilized by ultraviolet irradiation, and then washed with ethanol. Mice were anesthetized using isoflurane (Wako, Osaka, Japan) and then sterilized Ni wires were implanted subcutaneously in their dorsa using a 13 G implant needle (Natsume, Tokyo, Japan). In the control group, mice underwent a similar surgical procedure, but without the implantation of the Ni wire.

### ELISA

After incubation of each of the sample, IL-8 in the supernatants was assayed using an ELISA kit (eBioscience, San Diego, CA) according to the manufacturer’s protocol.

### Real-time PCR

Total RNA was extracted from the mouse skin tissue surrounding the Ni wire using RNAiso Plus (Takara, Shiga, Japan) according to the manufacturer’s protocol. The total RNA was reverse-transcribed into complementary DNA (cDNA) using the PrimeScript RT reagent kit (Takara, Shiga, Japan). Subsequently, real-time PCR was performed using an SYBR^®^ Premix Ex Taq^TM^ II (Takara, Shiga, Japan) and the Takara PCR Thermal Cycler Dice^®^ real time system (TP800, Takara, Shiga, Japan). The oligonucleotides used for RT-PCR were the following: Mouse GAPDH: (forward) 5′-TGT GTC CGT CGT GGA TCT GA-3′ and (reverse) 5′-TTG CTG TTG AAG TCG CAG GAG-3′, mouse MIP-2: (forward) 5′-CCA CCA ACC ACC AGG CTA CAG GGG C-3′ and (reverse) 5′-AGC CTC CTC CTT TCC AGG TCA GTT AGC-3′, mouse COX-2: (forward) 5′-GAA GTC TTT GGT CTG GTG CCT G-3′ and (reverse) 5′-GTC TGC TGG TTT GGA ATA GTT GC-3′. The normalization and fold changes were calculated using the ΔΔC_t_ method.

### Determination of Ni^2+^, Zn^2+^, Mn^2+^, Co^2+^ concentrations with ICP-MS

THP-1 cells were stimulated by NiCl_2_ for 24 h in Fig. 1a and b, or for the indicated time in Fig. 1c and d. The cells were stimulated by NiCl_2_ and/or other metal chlorides for 24 h in Figs 2, 4 and 5. After the incubation, they were collected and washed five times with PBS (phosphate-buffered saline), and then suspended in 150 μl PBS. The cell suspension was sonicated for 30 s and the aliquot was diluted 10-fold with 5% (w/w) HNO_3_. The concentration of Ni^2+^ and other metal ions in each sample was determined by Agilent 7500 Series ICP-MS (Agilent Technology, Santa Clara, CA).

To determine the metal concentrations in the mouse skin and serum, circular skin tissue sections (1 cm in diameter) from the region surrounding the Ni wire were excised and the wet weight of skin was measured. The skin tissue sample, approximately 80 mg, was boiled in 3 ml 69% (w/w) HNO_3_ for 30 min, and then, 300 μl 30% (w/v) H_2_O_2_ was added to the samples, on ice. The skin samples were then boiled again for approximately 30 min, and pure water was added to attain a total weight of 10 g. Mouse blood was incubated for 12 h at 4 °C and then centrifuged at 1,200 × *g*, 4 °C for 30 min. The supernatant was diluted 10-fold with 5% (w/w) HNO_3_, and centrifuged at 500 × *g*, 4 °C for 5 min. The supernatant was collected. The Ni^2+^, Zn^2+^ concentration of each sample was also determined by ICP-MS.

### Bradford determination of protein concentration

The protein contents in the sonicates of cells were determined using the Bio-Rad Protein Assay Dye Reagent Concentrate (Bio-Rad, Tokyo, Japan), according to the manufacturer’s protocol.

### Newport green fluorescence staining of intracellular Ni ions

THP-1 cells were stimulated by NiCl_2_ and/or ZnCl_2_ for 24 h. After the incubation, the cells were collected and washed five times with 1 × PBS, and then treated for 30 min with 5 μM Newport Green^TM^ DCF diacetate (Invitrogen, Carlsbad, CA) dissolved in dimethyl sulfoxide. After this treatment, the cells were washed once with 1 × PBS and placed on a Micro Slide Glass (76 × 26 mm, 0.9–1.2 mm thickness, Matsunami-glass, Osaka, Japan), cover-slipped with Fluoromount (DBS, Diagnostic BioSystems, CA). Fluorescence images (excitation at 505 nm and emission at 535 nm) were acquired using a laser scanning confocal microscope LSM 800 (Carl Zeiss, Germany).

### Statistical analysis

The statistical significance of the results was analyzed using the unpaired two-tailed Student’s *t*-test, and the Bonferroni multiple comparison test or Student-Newman-Keuls test for multiple comparisons. For some experiments, a statistical outlier removal was performed using the Smirnov-Grubbs’ rejection test and the Thompson test.

## References

[1] Gutensohn K (2000). *In vitro* analyses of diamond-like carbon coated stents. Reduction of metal ion release, platelet activation, and thrombogenicity. Thromb. res..

[2] Hirasawa N (2010). Involvement of prostaglandins and histamine in nickel wire-induced acute inflammation in mice. J. Biomed. Mater. Res..

[3] Wataha JC (2001). Relating nickel-induced tissue inflammation to nickel release *in vivo*. J. Biomed. Mater. Res..

[4] Fujiu K (2012). Nickel-free stainless steel avoids neointima formation following coronary stent implantation. Sci. Technol. Adv. Mater..

[5] Sato T (2016). Involvement of COX-2 in nickel elution from a wire implanted subcutaneously in mice. Toxicology..

[6] Kishimoto Y (2017). Induced histamine regulates Ni elution from an implanted Ni wire in mice by downregulating neutrophil migration. Exp. Dermatol..

[7] Gamerdinger K (2003). A new type of metal recognition by human T cells: contact residues for peptide-independent bridging of T cell receptor and major histocompatibility complex by nickel. J. Exp. Med..

[8] Lu L (2003). Components of the ligand for a Ni^++^ reactive human T cell clone. J. Exp. Med..

[9] Thierse HJ (2004). Metal-protein complex-mediated transport and delivery of Ni^2+^ to TCR/MHC contact sites in nickel-specific human T cell activation. J. Immunol..

[10] Jakob A (2017). Immunoproteomic identification and characterization of Ni^2+^-regulated proteins implicates Ni^2+^ in the induction of monocyte cell death. Cell Death Dis..

[11] Schmidt M (2010). Crucial role for human Toll-like receptor 4 in the development of contact allergy to nickel. Nat. Immunol..

[12] Ke Q, Costa M (2006). Hypoxia-inducible factor-1 (HIF-1). Mol. Pharmacol..

[13] Eitinger T, Mandrand-Berthelot MA (2000). Nickel transport systems in microorganisms. Arch. Microbiol..

[14] Stoof J, Kuipers EJ, Klaver G, van Vliet AH (2010). An ABC transporter and a TonB ortholog contribute to Helicobacter mustelae nickel and cobalt acquisition. Infect. Immun..

[15] Gunshin H (1997). Cloning and characterization of a mammalian proton-coupled metal-ion transporter. Nature..

[16] Kasprzak KS, Sunderman FW, Salnikow K (2003). Nickel carcinogenesis. Mutat. Res..

[17] Guerinot ML (2000). The ZIP family of metal transporters. Biochim. Biophys. Acta..

[18] Edwards DL, Wataha JC, Hanks CT (1998). Uptake and reversibility of uptake of nickel by human macrophages. J. Oral Rehabil..

[19] Li L (2013). Effect of Ni (II) on inflammatory gene expression in THP1 monocytic cells. J. Biomed. Mater. Res. A..

[20] Nukada Y (2008). Production of IL-8 in THP-1 cells following contact allergen stimulation via mitogen-activated protein kinase activation or tumor necrosis factor-alpha production. J. Toxicol. Sci..

[21] Thierse HJ, Helm S, Pink M, Weltzien HU (2007). Novel fluorescence assay for tracking molecular and cellular allergen-protein interactions. J. Immunol. Methods..

[22] Buckett PD, Wessling-Resnick M (2009). Small molecule inhibitors of divalent metal transporter-1. Am J Physiol Gastrointest Liver Physiol..

[23] Yanatori I, Yasui Y, Noguchi Y, Kishi F (2015). Inhibition of iron uptake by ferristatin II is exerted through internalization of DMT1 at the plasma membrane. Cell Biol. Int..

[24] Nebert DW (2012). ZIP14 and ZIP8 zinc/bicarbonate symporters in Xenopus oocytes: characterization of metal uptake and inhibition. Metallomics..

[25] Gaither LA, Eide DJ (2001). The human ZIP1 transporter mediates zinc uptake in human K562 erythroleukemia cells. J. Biol. Chem..

[26] Dufner-Beattie J, Langmade SJ, Wang F, Eide D, Andrews GK (2003). Structure, function, and regulation of a subfamily of mouse zinc transporter genes. J. Biol. Chem..

[27] Hamon R (2014). Zinc and zinc transporters in macrophages and their roles in efferocytosis in COPD. Plos One..

[28] Gaither LA, Eide DJ (2000). Functional expression of the human hZIP2 zinc transporter. J. Biol. Chem..

[29] Pinilla-Tenas JJ (2011). Zip14 is a complex broad-scope metal-ion transporter whose functional properties support roles in the cellular uptake of zinc and nontransferrin-bound iron. Am J Physiol Cell Physiol..

[30] Girijashanker K (2008). Slc39a14 gene encodes ZIP14, a metal/bicarbonate symporter: similarities to the ZIP8 transporter. Mol. Pharmacol..

[31] Liuzzi JP (2005). Interleukin-6 regulates the zinc transporter Zip14 in liver and contributes to the hypozincemia of the acute-phase response. Proc. Natl. Acad. Sci. USA.

[32] Yasuda H, Tsutsui T (2016). Infants and elderlies are susceptible to zinc deficiency. Sci. Rep..

